# Steam Explosion Pretreatment Changes Ruminal Fermentation *in vitro* of Corn Stover by Shifting Archaeal and Bacterial Community Structure

**DOI:** 10.3389/fmicb.2020.02027

**Published:** 2020-08-28

**Authors:** Kun Wang, Xuemei Nan, Jinjin Tong, Guangyong Zhao, Linshu Jiang, Benhai Xiong

**Affiliations:** ^1^State Key Laboratory of Animal Nutrition, Institute of Animal Science, Chinese Academy of Agricultural Sciences, Beijing, China; ^2^State Key Laboratory of Animal Nutrition, College of Animal Science and Technology, China Agricultural University, Beijing, China; ^3^Beijing Key Laboratory for Dairy Cow Nutrition, Beijing University of Agriculture, Beijing, China

**Keywords:** steam explosion, corn stover, ruminal fermentation *in vitro*, archaeal community, bacterial community

## Abstract

Steam explosion is an environment-friendly pretreatment method to improve the subsequent hydrolysis process of lignocellulosic biomass. Steam explosion pretreatment improved ruminal fermentation and changed fermentation pattern of corn stover during ruminal fermentation *in vitro*. The study gave a comprehensive insight into how stream explosion pretreatment shifted archaeal and bacterial community structure to change ruminal fermentation *in vitro* of corn stover. Results showed that steam explosion pretreatment dramatically improved the apparent disappearance of dry matter (DM), neutral detergent fiber (NDF), and acid detergent fiber (ADF). Steam explosion pretreatment significantly increased the molar proportion of propionate and decreased the ratio of acetate to propionate. At archaeal level, steam explosion pretreatment significantly increased the relative abundance of *Methanobrevibacter*, which can effectively remove metabolic hydrogen to keep the fermentation continuing. At bacterial level, the shift in fermentation was achieved by increasing the relative abundance of cellulolytic bacteria and propionate-related bacteria, including Spirochaetes, Elusimicrobia, Fibrobacteres, *Prevotella*, *Treponema*, *Ruminococcus*, and *Fibrobacter*.

## Introduction

Corn stover is an abundant agricultural residue that can be harvested as a ruminant feedstuff or used to produce biofuel. As a lignocellulosic material, corn stover is mainly composed of three types of polymers: cellulose, hemicellulose, and lignin. These three polymers form a complex network to resist the degradation by microorganisms and enzymes (Malinovsky et al., 2014). Several physical and chemical pretreatment methods have been applied to enhance the utilization of lignocellulosic materials, such as grinding, acid, alkali, enzyme, and steam explosion pretreatment (Schumacher et al., 2014; Schroyen et al., 2015; Bolado-Rodriguez et al., 2016). Although chemical pretreatment methods are useful strategies to improve the utilization of lignocellulosic materials, the chemicals used during processes must be removed and environmental pollution also follows these methods. Compared to chemical pretreatment methods, steam explosion has been recognized as an environment-friendly pretreatment and broadly used for numerous lignocellulosic biomass such as corn stover (Zhao et al., 2018), wheat straw (Ferreira et al., 2013), birch (Vivekanand et al., 2013), and *Salix* (Horn et al., 2011). Steam explosion is an explosion caused by violent boiling of water into steam, which heats the biomass at high temperature and rapidly drops pressure to disrupt the biomass fibers (Horn et al., 2011). Recent studies have shown that steam explosion can effectively improve digestibility of lignocellulosic biomass (Zhao et al., 2018) and increase the methane (CH_4_) production during anaerobic digestion (Lizasoain et al., 2017). Steam explosion mainly reduces cellulose degree of polymerization to enhance the digestibility of lignocellulosic biomass (Yu et al., 2015; Jin et al., 2016).

The rumen is a fermentation vat that is home to a vast array of bacteria, protozoa, fungi, and archaea. The structure of ruminal microbiomes is closely related to the process of rumen microbial fermentation. The microorganisms in rumen have been found to be able to effectively ferment lignocellulosic biomass into chemical compounds (Jami et al., 2013; Yue et al., 2013). Volatile fatty acids (VFA) and CH_4_ are the two major products in rumen microbial fermentation process. VFA will be subsequently absorbed by ruminants to convert into food products (Jami et al., 2013); While, CH_4_ will be released to atmosphere and presents a loss of gross energy intake (2–12%), depending on types of diets (Johnson and Johnson, 1995). In the typical ruminal microbial fermentation process, the formation of acetate and butyrate both result in the release of metabolic hydrogen which must then be re-oxidized to continue the fermentation process (Ungerfeld, 2015). Methanogenic archaea are the only producer of CH_4_ in rumen and methanogenesis is the main route to remove metabolic hydrogen which is transferred from bacteria, protozoa, and fungi to archaea (Wolin and Miller, 1997). Steam explosion pretreatment could lead to increased digestibility and metabolic hydrogen production, usually accompanied by CH_4_ production. Propionate formation is also considered as an alternative pathway to utilize metabolic hydrogen in rumen (Moss et al., 2000). The strong negative correlation between propionate formation and CH_4_ formation has been proved by Pinares-Patino et al. (2003) and Goopy et al. (2006). Ungerfeld (2015) reported that inhibition of methanogenesis increased the propionate production by redirecting the flow of metabolic hydrogen.

Zhao et al. (2018) reported that steam explosion pretreatment increased digestibility and fermentation of corn stover by improving ruminal microbial colonization. Du et al. (2019) reported that stream explosion pretreatment significantly altered the fermentation parameters *in vitro* of wheat straw and decreased the ratio of acetate to propionate by facilitating sugar production and microbial colonization. However, it is unknown whether stream explosion pretreatment shifts the structure of ruminal microbiomes to improve fermentation and change fermentation pattern. The objective of this study was to explore how steam explosion pretreatment shifts archaeal and bacterial community structure to enhance fermentation of corn stover and change ruminal fermentation pattern by building a model of corn stover and steam-exploded corn stover ruminal fermentation *in vitro*.

## Materials and Methods

### Experimental Design

Corn stover used in present study was harvested in a cornland at Yanqin District Beijing, China. The stover was left and dried in the field after harvest for about 2 months. The full stalk was cut into 50 cm fragments to make it easy to carry. The stover was dried at 65°C for 24 h to obtain air-dried samples. The air-dried corn stover was manually chopped to 3–5 cm fragments by scissors. One 500 g of (dry matter, DM) chopped corn stover samples was milled to pass through a 1 mm sieve using a Wiley mill (Thomas Scientific, Swedesboro, NJ, United States) as the control group fermentation substrate. A 20 L steam explosion reactor connected to a steam generator (QB-200, Hebi Gentle Bioenergy Co. Ltd., China) was used to produce steam-exploded corn stover. Another 500 g of (DM) chopped corn stover samples was accurately weighed, and then atomized evenly 50 g of deionized water on the samples. The samples were sealed in a vacuum bag and infiltrated for about 12 h at room temperature. The steam explosion parameters selected in present study were at 1.5 MPa steam pressure and 10% moisture for 3 min. The steam-exploded corn stover was oven-dried at 65°C for 24 h and then used for the treatment group fermentation substrate. A completely randomized design was applied in an *in vitro* incubation, and there are two treatments in the current study: CON (the control group fermentation substrate, corn stover) and TRT (the treatment group fermentation substrate, steam-exploded corn stover).

The experimental procedures were approved by the Chinese Academy of Agricultural Sciences Animal Care and Use Committee (Beijing, China). Rumen fluid was collected 2 h before morning feeding from three cannulated lactating Holstein cows fed a total mixed ration (TMR) composed (% DM basis) of corn silage (25%), alfalfa hay (15%), steam-flaked corn (27%), soybean meal (8.5%), cottonseed meal (8.5%), beet pulp (5.5%), distillers dried grains with solubles (7.5%), minerals, and vitamins (3%). The donor cows were fed twice daily at 0500 and 1700 h. Ruminal fluid was filtered through four layers of cheesecloth and immediately brought to the laboratory. Rumen fluid sampled from each of the donor cows was combined on an equal volume basis and diluted with buffer solution (1:2 v/v) formulated as the method of Menke (1988), containing per liter 8.75 g NaHCO_3_, 1.00 g NH_4_HCO_3_, 1.43 g Na_2_HPO_4_, 1.55 g KH_2_PO_4_, 0.15 g MgSO_4_·7H_2_O, 0.52 g Na_2_S, 0.017 g CaCl·2H_2_O, 0.015 g MnCl_2_·4H_2_O, 0.002 g CoCl·6H_2_O, 0.012 g FeCl_3_·6H_2_O, and 1.25 mg resazurin. The preparation process of the mixed liquid is carried out under continuous flushing with carbon dioxide (CO_2_). The starting pH of the mixed liquid after equilibration under CO_2_ is 6.80. The mixed fluid was transferred into 120-ml serum bottles (75 ml/bottle) containing 500 mg of fermentation substrates (CON or TRT) under the continuous flow of CO_2_ to remove the air from headspace. In addition, an extra four bottles containing no substrate served as blank for correction of analytes and gases. The serum bottles were immediately sealed with butyl rubber stoppers plus aluminum caps and then connected with vacuumed air bags. The batch fermentation was incubated at 39°C for 72 h with horizontal shaking at 60 rpm. The *in vitro* batch fermentation was carried out in triplicates and four serum bottles per treatment were arranged in each batch.

### Sample Collection and Analysis

The 100-ml calibrated glass syringes (Häberle Labortechnik, Lonsee-Ettlenschieß, Germany) were used to measure the total gas production of each air bag. The pH of incubations was measured using a SevenGo™ portable pH meter (Mettler Toledo, Switzerland). The pre-weighed nylon bags (8 cm × 12 cm, 42 μm) were used to filter the whole biomass material of each bottle. Then, the nylon bags were washed with cold running water until the effluent ran clear. Afterwards, the nylon bags were dried at 55°C for 48 h for analysis of the apparent disappearance of DM, neutral detergent fiber (NDF), and acid detergent fiber (ADF). The filtrate samples (2.5 ml) from each bottle were individually collected for determining VFA profiles, and another 2.5 ml of filtrate sample was collected for microbial analysis. The samples for VFA profiles were frozen at −20°C and the samples for microbial analysis were frozen immediately in liquid nitrogen. The ANKOM A200 fiber analyzer (ANKOM Technology, Macedon, NY, United States) was used to measure the contents of NDF and ADF according to the approach described by Van Soest et al. (1991). Sodium sulfite and α-amylase were used for the analysis of NDF. The concentrations of CH_4_ and hydrogen (H_2_) were determined using a gas chromatograph (7890B, Agilent Technologies, United States) fitted with a thermal conductivity detector and a packed column (Porapak Q, Agilent Technologies, United States). The VFA concentrations were analyzed by a gas chromatograph (7890B, Agilent Technologies, United States) equipped with a flame ionization detector and a capillary column (30 m × 0.250 mm × 0.25 μm; BD-FFAP, Agilent Technologies, United States). More detail methods were described by Wang et al. (2018).

### DNA Extraction, Microbial 16S RNA Genes Amplification and Sequencing

Microbial DNA was extracted using an E.Z.N.ATM Mag-Bind Soil DNA kit (Omega, Norcross, Georgia, United States), in accordance with the manufacturer’s instructions. The quality and concentration of the extracted DNA were assessed by 1% agarose gel electrophoresis and a Qubit 3.0 spectrometer (Invitrogen, United States), respectively. This method gave DNA yields sufficient for analysis (averaging 35 ng/μl), but kit-based assays may yield non-representative taxonomic sampling (Henderson et al., 2013).

The primers 515F (5'-GTGCCAGCMGCCGCGGTAA-3') and 806R (5'-GGACTACHVGGGTWTCTAAT-3') were selected for bacterial community analysis (Caporaso et al., 2011). The analysis of archaeal community was conducted by the nested PCR according to the lower abundance of archaeal community compared with bacterial community. The process of nested PCR was conducted using the primers Arch340F (5'-CCCTAYGGGGYGCASCAG-3')/Arch1000R (5'-GAGARGWRGTGCATGGCC-3') and Arch349F (5'-GYGCASCAGKCGMGAAW-3')/Arch806R (50-GGACTACVSGGGTATCTAAT-3'), as previously described by Wang et al. (2018). The 16S ribosomal RNA (rRNA) gene amplicons were pooled and sent to Shanghai Sangon Biotech Co., Ltd. for pair-end sequencing using the Illumina MiSeq platform (2 × 300 bp).

### Sequencing Data Processing and Analysis

Sequencing reads were matched to different samples in accordance with the unique barcode of different samples, and then pair-end reads were merged using FLASH (Magoč and Salzberg, 2011). The quality of these merged reads was controlled by using PRINSEQ (Schmieder and Edwards, 2011). Then, the barcode and primers were removed, and UCHIME was used to filter out PCR chimeras to get clean sequences (Edgar et al., 2011). After filtration, the average length of all the clean reads was 416 and 379 bp, and the average sequencing depth was ca. 58,574 and 61,208 clean reads for bacterial and archaeal community analysis, respectively. After removal of singletons, operational taxonomic units (OTU) were clustered at 97% sequence identity using UPARSE (Edgar et al., 2011). The taxonomic classification of the sequences was carried out using the ribosomal database project (RDP) classifier at the bootstrap cutoff of 80%, as suggested by the RDP. The alpha diversity indices including Simpson, Shannon, Chao1, Coverage, and ACE were calculated through the QIIME 2 software package. The principal coordinate analysis (PCoA) was conducted by the weighted UniFrac distance (Lozupone et al., 2007), and the significant difference between treatments was assessed by an analysis of similarity (ANOSIM) in QIIME with 999 permutations (R Core Team, 2013). The extended error bar plot was performed to visualize the difference in relative abundance of bacteria and archaea by bioinformatics software (STAMP; Parks et al., 2014).

All the raw sequences were submitted to the NCBI Sequence Read Archive (SRA; http://www.ncbi.nlm.nih.gov/Traces/sra/), under accession number SRP189861.

### Statistical Analysis

Data were analyzed using PROC MIXED of SAS (version 9.4; SAS Institute Inc., Cary, NC). Spearman’s rank correlations between the relative abundance of microbial genera and fermentation variables were analyzed using the PROC CORR procedure of SAS (version 9.4; SAS Institute Inc., Cary, NC). *p* < 0.05 was considered statistically significant.

## Results and Discussion

### Changes in Ruminal Fermentation *in vitro* in Response to Steam Explosion Pretreatment

Data of substrate apparent disappearance, the molar proportion of VFA, and CH_4_ and H_2_ production are presented in Table 1. Based on the previous studies on *in vitro* forage fiber digestion, we terminated the incubation process at 72 h (Cross et al., 1974; Zhao et al., 2018). Steam explosion pretreatment dramatically improved the apparent disappearance of DM, NDF, and ADF. This was similar to the previous study, which reported that the improvement in digestibility of the steam-exploded corn stover was consistent with the enhanced colonization of microorganism (Zhao et al., 2018). Some other studies also reported that steam explosion pretreatment can enhance the degradability of lignocellulosic biomass by increasing porosity (Dan et al., 2017) and reducing degree of polymerization (Jin et al., 2016). In response to the improved DM degradability, the concentration of total VFA was significantly increased, which was similar to the previous studies (Zhao et al., 2018; Du et al., 2019). Steam explosion pretreatment increased the release of sugars from corn stover to improve the production of VFA (Zhao et al., 2018; Du et al., 2019). Steam explosion pretreatment changed the fermentation pattern and promoted a shift in the fermentation pattern toward a higher molar proportion of propionate in the present study. Du et al. (2019) also reported that steam explosion pretreatment increased the molar proportion of propionate. Fermentation of nonstructural carbohydrates (i.e., sugars, starches, organic acids, and other reserve carbohydrates), compared to fermentation of structural carbohydrates (i.e., cellulose, hemicellulose, and lignin), increased amounts of propionate and decreased the ratio of acetate and propionate (Dijkstra, 1994). Therefore, the higher molar proportion of propionate should be attributed to more soluble nonstructural carbohydrates released from steam explosion pretreatment. Zhao et al. (2018) and Du et al. (2019) both reported that steam explosion pretreatment increased the release of soluble nonstructural carbohydrates. Corresponded with the increased total VFA concentration and the higher molar proportion of propionate, the pH of TRT was significantly decreased compared with CON. Previous studies also reported that increases in total VFA concentrations and the molar proportion of propionate were associated with the decrease in pH due to containing more soluble nonstructural carbohydrates in diets (Penner and Oba, 2009; Song et al., 2018). Total gas production and CH_4_ production from TRT were both higher than that from CON, but the increases were both statistically insignificant. The results are not consistent from previous studies (Lizasoain et al., 2017; Mulat et al., 2018), which reported that steam explosion pretreatment significantly increased the total gas production and CH_4_ production. Steam explosion pretreatment improved the digestibility of corn stover and more readily fermentable substrates should produce more gas. While the lack of significant increase in total gas production and CH_4_ production in the present study might be attributed to the limited number of substrates initially added in the *in vitro* incubation. In addition, the anaerobic digestion inoculum used in the present study is rumen fluid, which is home to a vast array of ciliate protozoa, fungi, bacteria, and archaea. The compositions and interactions of different microbes are more complex than those used in the previous studies (Lizasoain et al., 2017; Mulat et al., 2018). In the present study, the formation of propionate might be served as an alternative to H_2_ formation and then reduce the utilization of H_2_ in methanogenesis, shifting H_2_ flow from CH_4_ to propionate production. Zhang et al. (2016) reported that propionate accumulation led to lower CH_4_ production of microwave pretreatment food waste compared to microwave pretreatment sewage sludge. Therefore, the higher proportion of propionate or the changed fermentation pattern may limit the significant increase in CH_4_ production in the present study. In the present study, there was a 24% increase in DM disappearance from the TRT fermentation compared to the CON fermentation (from 61.9 to 76.7%), but only a 4.6% increase in VFA production (from 62.8 to 65.7 mM), and increases in gas production were not significant. Much more DM disappeared than was recovered in VFA production. The reason may be due to sugars being released, and not being fermented to VFA. Zhao et al. (2018) and Du et al. (2019) reported that the concentration of reducing sugar, xylose, and fructose in steam explosion group increased after the 72 h of incubation. The chemical composition of corn stover and steam-exploded corn stover was shown in Supplementary Material.

**Table 1:** Effects of steam explosion pretreatment on fermentation and gas production after 72 h of incubation (*n* = 12).

Items	Treatment^1^		SEM^2^	*p*
	CON	TRT		
pH	6.61	6.54	0.009	<0.001
Apparent disappearance of DM^3^, %	61.9	76.7	1.57	<0.001
Fiber digestibility
NDF^4^, %	49.0	61.8	1.45	<0.001
ADF^5^, %	53.8	68.4	1.58	<0.001
Total VFA^6^, mM	62.8	65.7	0.39	0.001
Individual, mol/100 mol
Acetate	67.1	66.2	0.07	<0.001
Propionate	20.9	22.9	0.15	<0.001
Acetate/propionate	3.21	2.89	0.024	<0.001
Total gas production/(ml)	40.5	45.5	2.15	0.253
H_2_/(ml)	0.13	0.13	0.129	0.391
CH_4_/(ml)	5.15	5.64	0.012	0.290

1 CON, the control group fermentation substrate, corn stover; TRT, the treatment group fermentation substrate, steam-exploded corn stover.

2 SEM, standard error of the mean.

3 DM, dry matter.

4 NDF, neutral detergent fiber.

5 ADF, acid detergent fiber.

6 VFA, valid fatty acid.

### Changes in Archaeal Community Structure in Response to Steam Explosion Pretreatment

**Figure 1:**
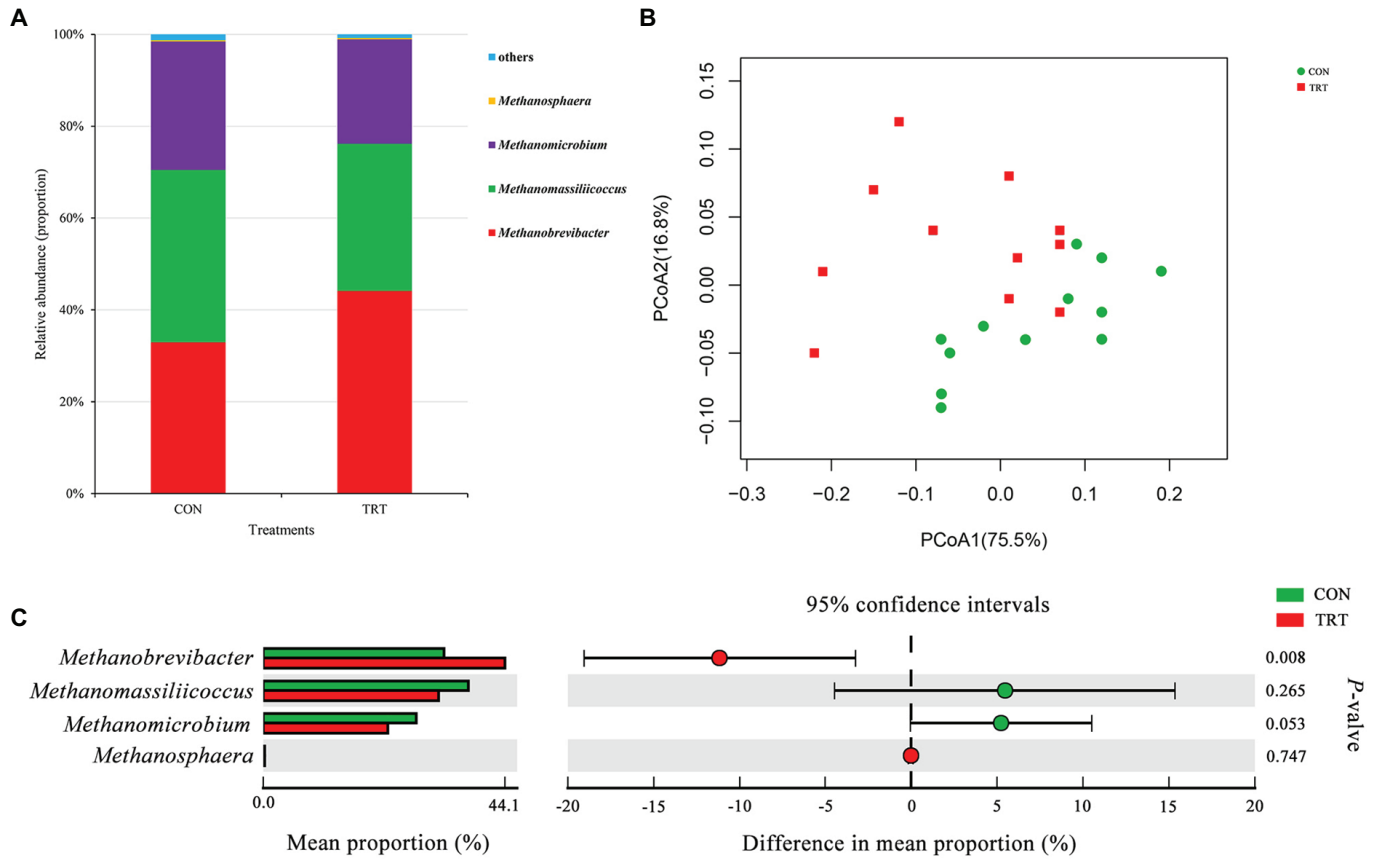
Changes in archaeal community structure. **(A)** The composition of the predominant archaeal genera. **(B)** Principal coordinate analysis (PCoA) of archaeal community structure. **(C)** Difference in the relative abundance of archaeal genera. CON, the control group fermentation substrate, corn stover; TRT, the treatment group fermentation substrate, steam-exploded corn stover (*n* = 12).

After merging and quality control, a total of 1,637,655 sequences from 24 samples were generated, and 1,572,671 high-quality sequences were acquired, with an average read length of 379 bp. After chimera removal, OTUs were obtained from the remaining 1,468,988 sequences, with 97% sequence similarity. After filtering, the remaining 24,035 OTUs were used for subsequent analysis. At archaeal phylum level, Euryarchaeota and Thaumarchaeota represented the archaeal community, where Euryarchaeota represented on average 99.99%. At archaeal genus level, *Methanobrevibacter* (38.53%), *Methanomassiliicoccus* (34.79%), *Methanomicrobium* (25.36%), and *Methanosphaera* (0.31%, Figure 1A) were the four predominant genera. *Methanobrevibacter* was recognized to be the most common genus in rumen (Janssen and Kirs, 2008; Danielsson et al., 2017). *Methanomassiliicoccus* has a high relative abundance similar to Methanobrevibacter in the present study.

Alpha archaeal diversity was presented in Table 2. No significant differences were observed between treatments based on the alpha diversity indices of Chao1, ACE, Shannon, and Simpson, showing that the archaeal community richness and diversity were not affected by steam explosion pretreatment. The analysis of PCoA based on the weighted UniFrac metrics (Figure 1B) showed that TRT was not distinctly separated from CON. Principal coordinate 1 and 2 accounted for 75.5 and 16.8% of the total variation, respectively. The ANOSIM analysis revealed a significant difference between CON and TRT (*R* = 0.125, *p* = 0.034).

**Table 2:** Alpha diversity indices of archaea and bacteria after 72 h of incubation (*n* = 12).

Items	Treatment^1^		SEM^2^	*p*
	CON	TRT		
Archaea
OTU^3^	997	1,007	23.2	0.828
Coverage	0.99	0.99	0.001	0.878
Chao1	1,983	1,959	39.0	0.765
ACE^4^	3,024	3,047	66.9	0.870
Shannon	2.26	2.22	0.027	0.524
Simpson	0.21	0.23	0.007	0.367
Bacteria
OTU	3,539	3,590	56.7	0.660
Coverage	0.97	0.97	0.001	0.960
Chao1	5,219	5,419	76.9	0.200
ACE	6,029	66,313	86.3	0.100
Shannon	6.34	6.40	0.015	0.031
Simpson	0.007	0.006	0.0002	0.018

1 CON, the control group fermentation substrate, corn stover; TRT, the treatment group fermentation substrate, steam-exploded corn stover.

2 SEM, standard error of the mean.

3 OTU, operational taxonomic units.

4 ACE, abundance-based coverage estimator.

At archaeal genus level, steam explosion pretreatment significantly increased the relative abundance of *Methanobrevibacter*. While the relative abundance of *Methanomassiliicoccus* and *Methanomicrobium* from TRT was lower than that from CON, the decreases were not statistically significant (Figure 1C). Spearman’s rank correlations between the relative abundance of archaeal genera and fermentation parameters were analyzed using the PROC CORR procedure of SAS 9.4 (Figure 3A). Within the archaeal community, no significant correlations were observed between the relative abundance of archaeal genera and CH_4_ production. As the sole producers of CH_4_ in rumen, the number of methanogens was considered to be related to CH_4_ production (Wallace et al., 2014; Veneman et al., 2015). However, the efficiency of different methanogens was considered to be more important than the number of methanogens in methanogenesis (Shi et al., 2014). Previous studies reported that *Methanobrevibacter* had positive correlations with CH_4_ production (Zhou et al., 2011; Danielsson et al., 2012; Wallace et al., 2015). Cunha et al. (2017) found that the relative abundance of *Methanosphaera* had negative correlations with CH_4_ production. There are two common methanogenic pathways in rumen: hydrogenotrophic pathway and methylotrophic pathway. Most methanogenic archaea use H_2_ and CO_2_ as substrates to produce CH_4_, but some species can also metabolize formate, methanol, or acetate for methanogenesis. *Methanobrevibacter* is the common hydrogenotrophic archaea, which produces 1 mole of CH_4_ for each mole of CO_2_ by hydrogenotrophic pathway (Hook et al., 2010), while *Methanosphaera* requires 4 moles of methanol to produce 3 moles of CH_4_ by methylotrophic pathway (Fricke et al., 2006). *Methanomassiliicoccus* has ability to use methylamine substrates to generate CH_4_ by H_2_-dependent methylotrophic pathway (Moissl-Eichinger et al., 2018). Methanogenic archaea commonly acquired substrates from environment, while some species would improve efficiency by building contacts with protozoa, which generated large amounts of H_2_ using hydrogenosomes (Embley et al., 2003). In rumen, *Methanobrevibacter* is recognized as the most common protozoa-associated methanogens, and the contribution of *Methanomicrobium* to protozoa-associated methanogenic community is different among studies (Belanche et al., 2014). In the present study, the improvement of corn stover in fermentation efficiency may be related to changes in archaeal community structure. Steam explosion mainly reduces cellulose degree of polymerization to enhance the digestibility of lignocellulosic biomass (Yu et al., 2015; Jin et al., 2016). Steam explosion pretreatment also increased the release of soluble nonstructural carbohydrates to improve the fermentation efficiency of corn stover (Zhao et al., 2018; Du et al., 2019). During the incubation *in vitro*, cellulose, hemicellulose, and soluble nonstructural carbohydrates are hydrolyzed to glucose and other monosaccharides, which are further metabolized to VFAs, CO_2_, and metabolic hydrogen (Beauchemin et al., 2020). The improvement of fermentation efficiency produced more fermentation products, including more metabolic hydrogen. Methanogenesis is the main route to remove metabolic hydrogen in rumen and *Methanobrevibacter* is an efficient hydrogenotrophic methanogenic archaea through the reduction of CO_2_ *via* H_2_ to produce CH_4_ (Hook et al., 2010). To keep the fermentation continuing, metabolic hydrogen must be removed to keep the partial pressure H_2_ low (Moss et al., 2000). Therefore, steam explosion pretreatment increased the relative abundance of *Methanobrevibacter* to enhance fermentation efficiency of corn stover.

### Changes in Bacterial Community Structure in Response to Steam Explosion Pretreatment

**Figure 2:**
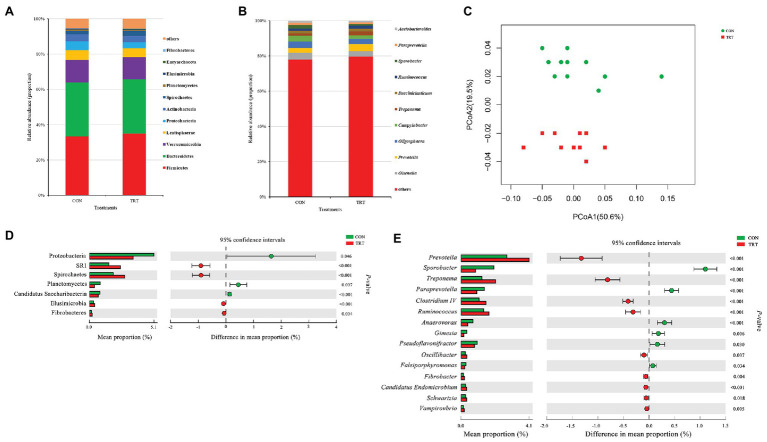
Changes in bacteria community structure. **(A)** The composition of the predominant bacterial phyla. **(B)** The composition of the predominant bacterial genera. **(C)** PCoA of bacterial community structure. **(D)** Difference in the relative abundance of bacterial phyla. **(E)** Difference in the relative abundance of bacterial genera. CON, the control group fermentation substrate, corn stover; TRT, the treatment group fermentation substrate, steam-exploded corn stover (*n* = 12).

After merging and quality control, a total of 1,543,897 sequences from 24 samples were generated, and 14,926,333 high-quality sequences were acquired, with an average read length of 416 bp. After chimera removal, OTUs were obtained from the remaining 1,405,778 sequences, with 97% sequence similarity. After filtering, the remaining 85,546 OTUs were used for subsequent analysis. At bacterial phylum level, *Firmicutes* and *Bacteroidetes* were the dominant phyla, representing 34.07 and 30.64% of the total sequences, respectively. Verrucomicrobia, Lentisphaerae, Proteobacteria, Actinobacteria, Spirochaetes, Planctomycetes, Elusimicrobia, Euryarchaeota, and Fibrobacteres represented average percentages of 12.78, 5.30, 4.25, 3.76, 2.33, 0.62, 0.36, 0.27, and 0.18%, respectively (Figure 2A). At bacterial genus level, the 10 predominant genera were *Olawnella* (3.48%), *Prevotella* (3.39%), *Oligosphaera* (3.35%), *Campylobacter* (2.69%), *Treponema* (1.65%), *Succiniclasticum* (1.51%), *Ruminococcus* (1.51%), *Sporobacter* (1.41%), *Paraprevotella* (1.17%), and *Acetobacteroides* (1.04%; Figure 2B).

Alpha bacterial diversity was presented in Table 2. No significant differences were observed between treatments based on Chao1 and ACE, showing that steam explosion pretreatment did not change the bacterial community richness. Changes of Shannon index and Simpson index indicated that steam explosion pretreatment significantly increased the diversity of bacterial community. The analysis of PCoA based on the weighted UniFrac metrics (Figure 2C) showed that TRT was significantly separated from CON. Principal coordinate 1 and 2 accounted for 50.6 and 19.5% of the total variation, respectively. The ANOSIM analysis revealed significant differences between treatments (*R* = 0.215, *p* = 0.001).

At bacterial phylum level, steam explosion pretreatment significantly increased the relative abundance of Spirochaetes, Elusimicrobia, and Fibrobacteres (Figure 2D). In rumen, Spirochetes can ferment xylan, pectin, and arabinogalactan, which are polymers commonly present in plant materials. Hydrolysis products of plant polymers (e.g., d-xylose, ʟ-arabinose, d-galacturonic acid, d-glucuronic acid, and cellobiose) also can be utilized as growth substrates by Spirochetes cultured from rumen fluid. Spirochetes fermented glucose to formate and acetate, some of them also produced succinate or ethanol (Paster and Canale-Parola, 1982). Succinate is not the end product of rumen fermentation and it will be further metabolized to propionate by other rumen species (Scheifinger and Wolin, 1973). Some important species belong to Spirochaetes, such as *Treponema bryantii* and *Treponema saccharophilum*, which were engaged primarily in the fermentation of soluble carbohydrates (Stanton and Canale-Parola, 1980). Steam explosion pretreatment destructed the integrity of cover stover and increased the release of sugars (Zhao et al., 2018), which could be served as fermentation substrates to produce succinate by Spirochaetes and finally converted to propionate by other rumen species. In the present study, the increased relative abundance of Spirochaetes may be related to the higher proportion of propionate in TRT group. Elusimicrobia is an enigmatic bacterial phylum in the hindgut of insects, as well as in rumen where it occurs as symbionts of various flagellated protists or as free-living bacteria (Méheust et al., 2019). Cultivation and genome-based studies revealed that some species belong to Elusimicrobia are capable of glucose fermentation (Geissinger et al., 2009; Zheng et al., 2016). In the present study, the increase in the relative abundance of Elusimicrobia may be attributed to the more release of sugars of corn stover resulted from steam explosion pretreatment. Fibrobacteres is a small bacterial phylum which currently comprises sole cultured representative genus, *Fibrobacter*, and *Fibrobacter succinogenes* and *Fibrobacter intestinalis* are the two cultured species, which were considered as major bacterial degraders of lignocellulosic material in rumen (Ransom-Jones et al., 2014). Steam explosion pretreatment broke the bonds between lignin and hemicellulose and cellulose, and damaged the surface structure of corn stover, so that fibrotic microbes had better interaction with lignocellulosic material (Zhao et al., 2018). More digestible lignocellulosic material may result in the increased relative abundance of Fibrobacteres.

**Figure 3:**
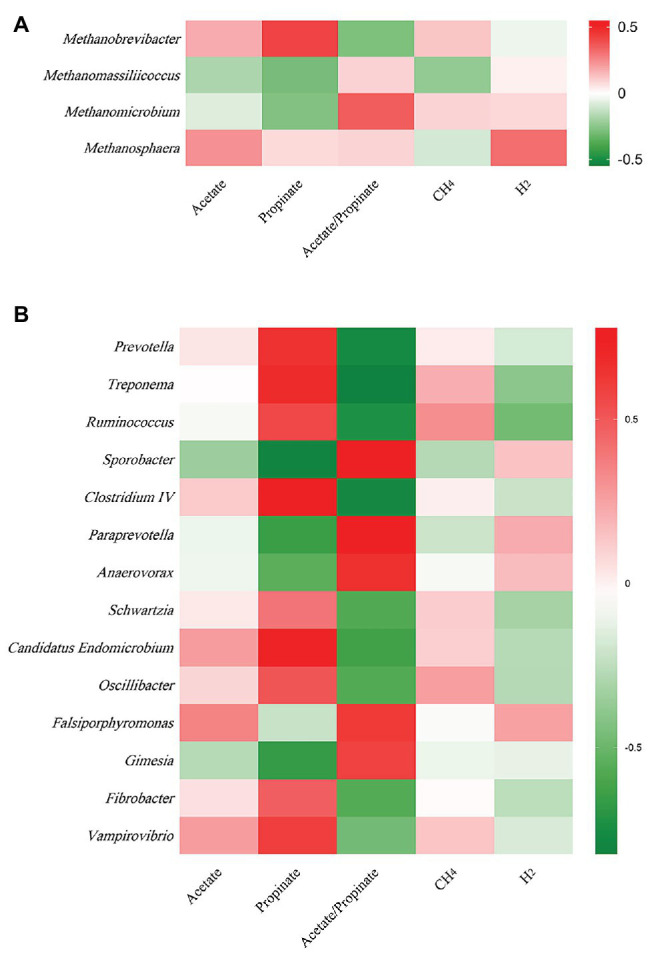
Spearman’s rank correlation between the relative abundances of archaeal and bacterial genera and fermentation parameters. **(A)** Between archaeal genera and fermentation parameters. **(B)** Between bacteria genera and fermentation parameters (*n* = 12).

At bacterial genus level, steam explosion pretreatment significantly increased the relative abundance of *Prevotella*, *Treponema*, *Ruminococcus*, *Oscillibacter*, *Fibrobacter*, and *Schwartzia* (Figure 2E). Spearman’s rank correlations indicated that the relative abundance of *Prevotella*, *Treponema*, and *Ruminococcus* has significantly positive correlations with propionate proportion (*r* > 0.55, *p* < 0.05) (Figure 3B). *Prevotella* is considered as the most abundant genus in rumen (de Menezes et al., 2011; Jami and Mizrahi, 2012) and one of the main propionate producers (Strobel, 1992). Different *Prevotella* species produced different fermentation end products, including that *Prevotella albensis* produced acetate, *Prevotella brevis* and *Prevotella bryantii* primarily produced propionate, and *P. ruminicola* produced propionate and acetate (Emerson and Weimer, 2017). *Treponema* is typically associated with the fermentation of soluble carbohydrates (Stanton and Canale-Parola, 1980). *Treponema* belongs to the phylum of Spirochaetes, which may be related to the higher proportion of propionate in the present study, as we talked about above. In addition, positive interactions have been demonstrated between *T. bryantii* and rumen cellulolytic bacteria (Kudo et al., 1987). *Ruminococcus* plays crucial roles in the fermentation of dietary polysaccharides in rumen (Leschine, 1995). Some *Ruminococcus* species are cellulolytic, including *Ruminococcus flavefaciens* and *Ruminococcus albus*, both of which were originally isolated from the bovine rumen (La Reau and Suen, 2018). Some *Ruminococcus* species are non-cellulolytic, including *Ruminococcus callidus* and *Ruminococcus bicirculans*. *R. callidus* can ferment saccharides and its major fermentation products are succinate and acetate, with a less amount of formate, lactate, and pyruvate (La Reau and Suen, 2018). Some of *Ruminococcus* can ferment polysaccharides to produce succinate, which will be further metabolized to propionate by other rumen species (Scheifinger and Wolin, 1973). At bacterial level, the improvement in fermentation efficiency and the shift in fermentation pattern may be achieved by increasing the relative abundance of cellulolytic bacteria and propionate-related bacteria. However, only filtrates were used for DNA isolation, so only the non-adherent (planktonic) community was analyzed in the present study. This community would be expected to be under-represented with respect to cellulolytic microbes when comparing the microbial data to fiber digestibility. Therefore, the potential bias associated with under-sampling the particle-associated community inevitably existed in the present study.

## Conclusion

Steam explosion pretreatment improved the fermentation efficiency and changed the fermentation pattern of corn stover during ruminal fermentation *in vitro* by shifting archaeal and bacterial community structure. Analysis of archaeal community structure revealed that steam explosion pretreatment significantly increased the relative abundance of *Methanobrevibacter*, which can effectively remove metabolic hydrogen to keep the fermentation continuing. At bacterial level, steam explosion pretreatment significantly increased the relative abundance of cellulolytic bacteria, including Fibrobacteres, *Fibrobacter*, and *Ruminococcus*. Steam explosion pretreatment significantly increased the relative abundance of propionate-related bacteria, including Spirochaetes, Elusimicrobia, *Prevotella*, *Treponema*, and *Ruminococcus*.

## Data Availability Statement

The datasets generated for this study can be found in the NCBI Sequence Read Archive/accession number SRP189861.

## Ethics Statement

The experimental procedures were approved by the Chinese Academy of Agricultural Sciences Animal Care and Use Committee (Beijing, China).

## Author Contributions

KW, BX, and LJ designed the study. KW and XN conducted the experiment and analyzed the data. KW wrote the manuscript. JT, XN, GZ, LJ, and BX revised the paper. All authors contributed to the article and approved the submitted version.

### Conflict of Interest

The authors declare that the research was conducted in the absence of any commercial or financial relationships that could be construed as a potential conflict of interest.

## Supplementary Material

The Supplementary Material for this article can be found online at: https://www.frontiersin.org/article/10.3389/fmicb.2020.02027/full#supplementary-material
