# Supplementary material for: Steam Explosion Pretreatment Changes Ruminal Fermentation in vitro of Corn Stover by Shifting Archaeal and Bacterial Community Structure
Source: Front Microbiol. 2020 Aug 28;11:2027. doi: 10.3389/fmicb.2020.02027 (PMC7483759; doi:10.3389/fmicb.2020.02027)
Supplement: Supplementary file 1 [file Table_1.docx]

Supplementary data

| Chemical components | Treatments^1^ | | SEM^2^ | *P* |
| --- | --- | --- | --- | --- |
|  | CON | TRT |  |  |
| NDF^3^ (%) | 68.4 | 50.6 | 0.25 | <0.001 |
| ADF^4^ (%) | 33.4 | 43.6 | 0.28 | <0.001 |
| NDS^5^(%) | 31.7 | 49.5 | 0.26 | <0.001 |
| Hemicellulose (%) | 34.9 | 6.9 | 0.21 | <0.001 |
| Cellulose (%) | 30.2 | 36.3 | 0.32 | 0.032 |
| Lignin (%) | 3.2 | 7.4 | 0.41 | 0.021 |

Table 1 The chemical composition of corn stover and steam-exploded corn stover

^1^CON, the control group fermentation substrate, corn stover; TRT, the treatment group fermentation substrate, steam-exploded corn stover.

^2^SEM, standard error of the mean.

^3^NDF, neutral detergent fiber.

^4^ADF, acid detergent fiber.

^5^NDS, neutral detergent solute.

The contents of NDF, ADF, and lignin were determined by the method of Van Soest et al. (1991). The content of hemicellulose was equal to the difference between NDF and ADF and that of cellulose was the difference between ADF and lignin. The NDS was obtained by subtracting the value of NDF on DM basis from DM of the respective corn stovers.

Van Soest, P.v., Robertson, J., Lewis, B. 1991. Methods for dietary fiber, neutral detergent fiber, and nonstarch polysaccharides in relation to animal nutrition. *Journal of dairy science*, **74**(10), 3583-3597.
